# Alterations in the composition of meibomian gland secretions in patients with meibomian gland dysfunction based on Raman spectroscopy

**DOI:** 10.3389/fmed.2025.1717118

**Published:** 2025-12-10

**Authors:** Shangkun Ou, Lingli Zhang, Yiming Wu, Dan Yang, Naikun Jiang, Ting Mao, Xueer Zheng, Hao Gu, Liying Zhang

**Affiliations:** 1 Department of Ophthalmology, The Affiliated Hospital of Guizhou Medical University, Guiyang, Guizhou, China; 2 Guizhou Medical University, Guiyang, Guizhou, China; 3 Department of Ophthalmology, The Qinglong County People's Hospital, Qinglong, Guizhou, China; 4 Xiamen University Affiliated Xiamen Eye Center, Fujian Provincial Key Laboratory of Ophthalmology and Visual Science, Fujian Engineering and Research Center of Eye Regenerative Medicine, Eye Institute of Xiamen University, School of Medicine, Xiamen University, Xiamen, Fujian, China; 5 School of Biomedical Sciences, Institute of Clinical Sciences, University of Birmingham, Birmingham, United Kingdom

**Keywords:** dry eye disease, fatty acids, lipids, meibomian gland, meibomian gland dysfunction, meibum, protein, Raman spectroscopy

## Abstract

**Purpose:**

To investigate biochemical alterations in meibum from patients with obstructive meibomian gland dysfunction (MGD) using Raman spectroscopy, with a focus on lipid saturation, molecular conformation, and lipid–protein composition.

**Methods:**

Meibum samples from healthy individuals and MGD patients with obstructive MGD were collected by eyelid expression, smeared onto gold-coated silicon slides, stored at low temperature, and analyzed within 2 h. Raman band-intensity ratios were used to quantify lipid unsaturation (I1659/I1438, I3013/I2860), lipid molecular conformation (I2888/I2860), and lipid-to-protein balance (I1659/I1161, I3013/I1161). Raman mapping was used to assess spatial distribution of biochemical components.

**Results:**

Meibum was lipid-rich in both groups. Ratios associated with lipid unsaturation (I1659/I1438 and I3013/I2860) were significantly lower in MGD samples than in healthy controls, indicating reduced unsaturated lipids. Ratios reflecting lipid-to-protein balance (I1659/I1161 and I3013/I1161) were significantly higher in MGD, suggesting a relative reduction in protein content. The symmetric-to-asymmetric lipid ratio (I2888/I2860) also differed significantly between groups, though with a small absolute effect size. Raman mapping further revealed a heterogeneous spatial distribution of lipids and proteins in MGD meibum.

**Conclusion:**

Compared with healthy meibum, MGD meibum exhibits reduced unsaturated lipid content, altered lipid-protein balance, and increased biochemical heterogeneity, accompanied by subtle conformational lipid changes. These findings indicate that both lipid composition and microstructural organization are disrupted in MGD. This study supports the potential of Raman spectroscopy serve as a valuable tool for analyzing meibum composition and detecting pathological changes in MGD.

## Introduction

The meibomian gland is a specialized sebaceous gland located in the eyelids that secretes lipids, which are the major components of the tear film lipid layer. These lipids are essential for maintaining ocular surface health and integrity. The lipid layer lubricates the ocular surface, reduces friction between the eyelids during blinking, prevents tear evaporation, stabilizes the tear film by lowering surface tension, and contributes to the refractive properties of the eye (1–5). Meibomian gland dysfunction (MGD) is a chronic, diffuse condition primarily characterized by obstruction of the terminal ducts and/or qualitative or quantitative abnormalities in meibum secretion. Clinically, MGD can cause tear film instability and ocular inflammation, leading to symptoms of ocular irritation. In severe cases, it may result in corneal damage and impaired visual function (6–8).

Meibum is composed of diverse lipid and protein components, including wax esters (32.3%), cholesterol esters (27.3%), polar lipids (15%), diesters (7.7%), triglycerides (3.7%), free fatty acids (2%), and free cholesterol (1.6%). These lipids are secreted onto the ocular surface to form the tear film lipid layer, which is critical for tear film stability (9–11). Significant differences in meibum composition have been reported between healthy individuals and MGD patients. In addition, meibum contains more than 90 proteins, such as keratins (K1, 5, 6, 7, 9, 10, 13, and 16), lactoferrin, lipophilic proteins, lipocalin (12), phospholipid transfer protein, surfactant proteins (SP-B and SP-C), proteoglycans, epidermal growth factor receptor, cytochrome C, fascin-X adhesive protein *α*-3 chain (13), and lysozyme C (5, 12). These proteins contribute to tear film stability and ocular homeostasis. Keratins, in particular, have been extensively studied in the context of ocular disease pathophysiology. Excessive keratin in meibum may compromise tear film stability, shorten tear film break-up time, and contribute to dry eye disease (14). Moreover, proteins in meibum are implicated in regulating tear colloid osmotic pressure (15) and ocular immune functions, although studies in this area remain limited.

Building on these findings, recent studies have employed Raman spectroscopy to investigate the biochemical composition of meibomian gland secretions. For example, Lin et al. (16) demonstrated that picosecond coherent anti-Stokes Raman spectroscopic imaging combined with multivariate analysis can be used to assess lipid distribution and composition in tissues. Similarly, Oshima et al. (17), Paugh et al. (18), and Suhalim et al. (19) utilized Raman spectroscopy to analyze lipid and protein alterations in meibomian gland secretions. Therefore, the present study applied Raman spectroscopy to explore lipid and protein differences between normal and abnormal meibomian gland secretions, aiming to provide a reliable theoretical and experimental foundation for the clinical detection and diagnosis of MGD.

## Methods

### Sample collection and participants

A total of 40 meibomian gland secretion samples were collected, including 20 from healthy individuals (10 males and 10 females; mean age, 34 ± 13 years) and 20 from patients diagnosed with obstructive meibomian gland dysfunction (MGD) (10 males and 10 females; mean age, 37 ± 16 years). All participants provided written informed consent prior to sample collection. Healthy donors reported no ocular discomfort, underwent comprehensive ophthalmic examinations that revealed no abnormalities, and exhibited clear, liquid meibomian gland secretions. Patients with obstructive MGD were diagnosed based on 2023 Chinese expert consensus on meibomian gland dysfunction (20, 21). This study was approved by the Clinical Research Ethics Committee of the First Affiliated Hospital of Xiamen University (KY2016-001) (22).

### Sample collection and storage

Meibum was classified as normal (clear and transparent) or abnormal (granular or toothpaste-like in consistency). For collection, participants first received warm compresses at 45 °C to facilitate secretion. Expression and massage of the meibomian glands were performed by the same experienced clinician using a cotton swab. The secretions were collected from both upper and lower eyelids with a stainless-steel spatula. Meibum samples were immediately smeared onto gold-coated silicon slides, transferred into 1.5 mL centrifuge tubes, sealed to prevent oxidation, stored at low temperature, and subjected to Raman spectroscopy within 2 h of collection.

### Raman spectroscopy

Raman spectra were acquired using a Nanophoton Raman-11 system coupled with an upright microscope (Nikon Eclipse 90i). A 532 nm solid-state Nd-YAG laser served as the excitation source. The system employed a 600 grooves/mm grating and a thermoelectrically cooled charge-coupled device (CCD) detector (400 × 1,340 pixels). A 20 × microscope objective (numerical aperture 0.45; working distance 4.5 mm) was used for both Raman spectral acquisition and white-light imaging of the samples. The laser spot diameter was approximately 2 μm. Unless otherwise specified, the laser power and acquisition time were set to 20 mW and 20 s for Raman mapping, and 5 mW and 10 s for colloidal Raman spectra.

### Statistical analysis

Experimental data were statistically analyzed using the SPSS 29 software. Independent sample *t*-tests were used for inter-group comparisons, and statistical significance was set at *p* < 0.05.

## Results

### Collection of Raman spectra of normal and abnormal meibum

**Figure 1:**
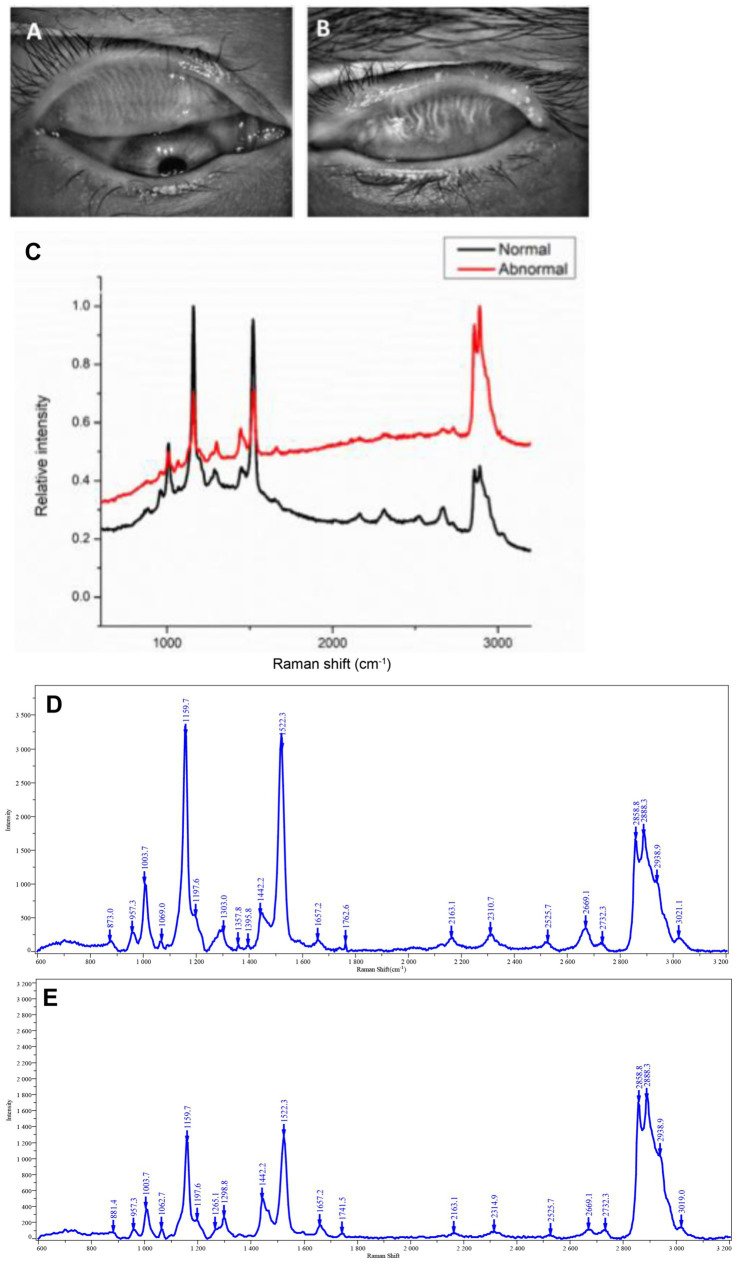
Raman spectra of meibum. **(A)** Infrared meibography of a normal meibomian gland. **(B)** Infrared meibography of a gland from an MGD patient. **(C)** Representative Raman spectra of meibum from healthy individuals and MGD patients. **(D)** Baseline-corrected Raman spectra of normal meibum. **(E)** Baseline-corrected Raman spectra of MGD meibum. All spectra were acquired under identical conditions.

Meibum samples from healthy individuals and MGD patients were collected, spread on gold-coated silicon slides, sealed, and analyzed within 2 h. Infrared meibography revealed intact, well-organized gland structures in healthy individuals (Figure 1A), whereas patients with MGD exhibited gland dropout (approximately grade 2 in the upper eyelid), distortion, and visible obstruction of gland orifices by meibum plugs (Figure 1B). Upon expression, clear and transparent meibum was observed in healthy donors, while secretions from MGD patients appeared turbid, granular, or toothpaste-like in consistency.

Raman spectroscopy was performed on both normal and abnormal meibum. For each sample, spectra were acquired from 20 points and averaged. The Raman peak positions were comparable between the two groups, but differences were observed in peak intensities (Figure 1C). After baseline correction (Figure 1D (healthy individuals) and Figure 1E (patients with MGD)), the Raman signal intensities at 1190 cm^−1^ and 1,522 cm^−1^ were consistently higher in normal meibum than in MGD samples.

**Figure 2:**
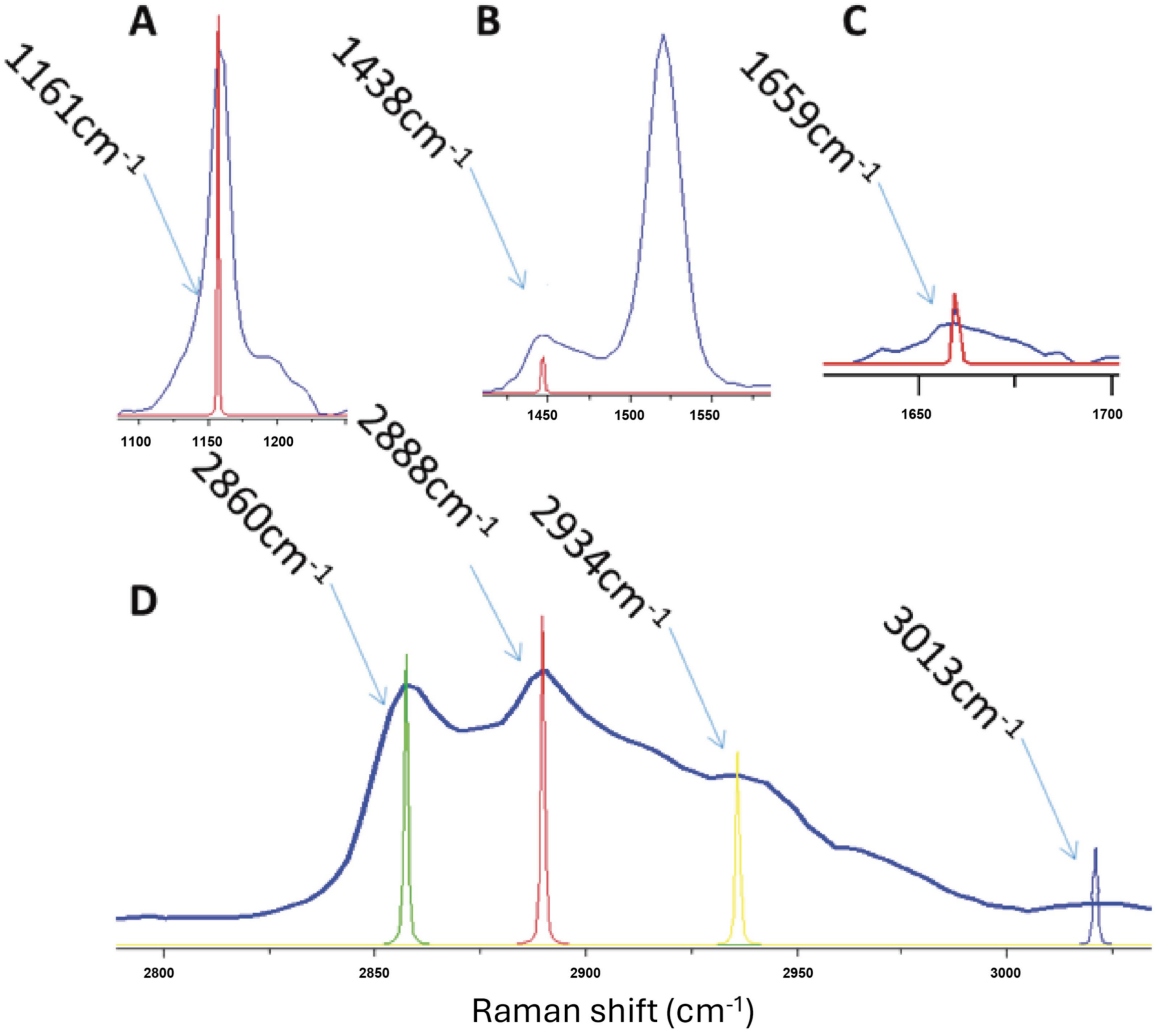
Raman band assignments of meibum. **(A)** 1,161 cm^−1^ and **(D)** 2,934 cm^−1^ correspond to proteins; **(B)** 1,438 cm^−1^, **(C)** 1,659 cm^−1^, and **(D)** 2,860, 2,888, and 3,013 cm^−1^ correspond to lipids.

Based on published literature (11, 17, 23, 24), Raman spectral peaks were assigned to protein and lipid components (Table 1). Peaks at 1161 cm^−1^ (Figure 2A) and 2,934 cm^−1^ (Figure 2D) correspond to proteins. Lipid-related peaks included 1,438 cm^−1^ (Figure 2B), 1,659 cm^−1^ (Figure 2C), and 2,860, 2,888, and 3,013 cm^−1^ (Figure 2D). Among these, 1,438, 2,860, and 2,888 cm^−1^ represent saturated lipids (primarily saturated fatty acids), whereas 1,659 and 3,013 cm^−1^ represent unsaturated lipids (primarily unsaturated fatty acids). The 2,860 cm^−1^ and 2,888 cm^−1^ peaks further correspond to symmetric and asymmetric lipid stretching vibrations, respectively.

**Table 1:** Raman band assignments of meibum based on previous reports.

Peak No.	Raman feature (cm^−1^)	Suggested assignment		
		DNA/RNA	Proteins	Lipids
1	1,161	G, C	C-H Tyr, Phe	
2	1,438			C-H band
3	1,659			cis C=C stretch
4	2,860			CH_2_ symmetric stretch
5	2,888			CH_2_ asymmetric stretch
6	2,934		The CH_2_ stretching vibration of proteins	
7	3,013			Stretching vibrations of C=C in unsaturated lipids

### Changes in the relative content of saturated and unsaturated lipids in MGD

Healthy meibum appears clear and transparent with good flowability, whereas meibum from patients with MGD is often granular, sandy, or toothpaste-like in consistency, with reduced flowability that requires heat application and moderate pressure for expression. Previous studies have demonstrated a strong correlation between lipid layer fluidity and the relative contents of saturated and unsaturated fatty acids. A higher proportion of unsaturated fatty acids increases lipid fluidity due to their lower melting points and higher mobility. Moreover, unsaturated fatty acids contribute to the regulation of inflammation and immune responses.

**Figure 3:**
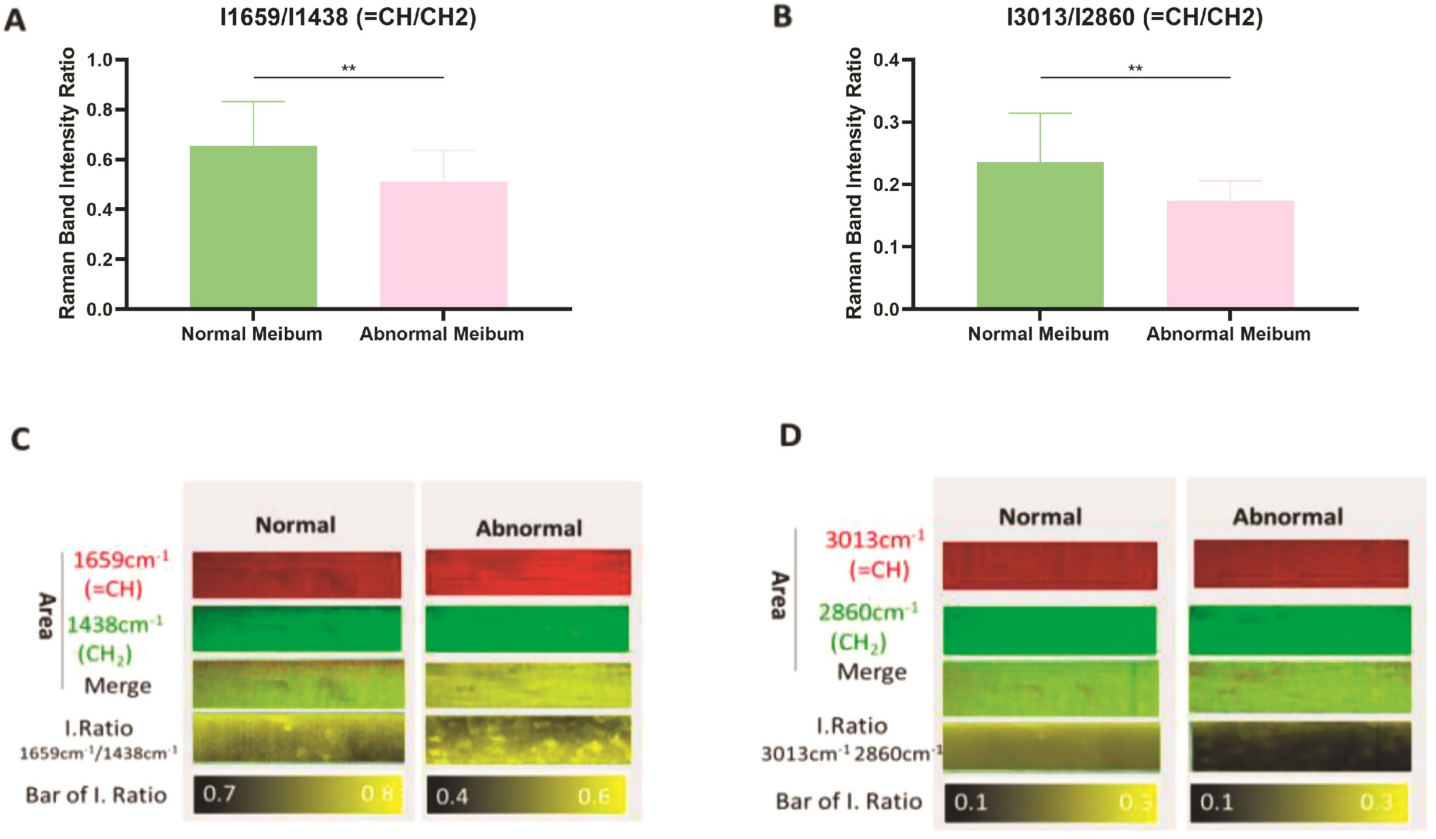
Assessment of lipid saturation in meibum using Raman spectroscopy. **(A)** Ratio of Raman band intensities I1659/I1438 cm^−1^ (unsaturated/saturated lipids) in normal and abnormal meibum. Data are presented as mean ± SD. Independent-samples *t*-test: Normal meibum (*n* = 20), 0.653497 ± 0.1779; abnormal meibum (*n* = 20), 0.512422 ± 0.1235; *p* = 0.0060 (*p* < 0.01). **(B)** Ratio of Raman band intensities I3013/I2860 cm^−1^ (unsaturated/saturated lipids) in normal and abnormal meibum. Data are presented as mean ± SD. Independent-samples *t*-test: Normal meibum (*n* = 20), 0.236362 ± 0.0777; abnormal meibum (*n* = 20), 0.173737 ± 0.0323; *p* = 0.0019 (*p* < 0.01). **(C,D)** Raman mapping analysis showing the spatial distribution of saturated and unsaturated lipids in normal and MGD meibum.

To investigate lipid composition in MGD, Raman spectroscopy was used to analyze the molecular structure of meibum and assess the relative content of saturated and unsaturated lipids. Lipid unsaturation was evaluated by calculating the ratios of Raman peak intensities corresponding to unsaturated lipids (1,659 cm^--1^ and 3,013 cm^−1^) to those of saturated lipids (1,438 cm^−1^ and 2,860 cm^−1^), expressed as I1659/I1438 and I3013/I2860. The unsaturation ratios were significantly higher in healthy meibum compared with MGD samples (*p* < 0.01). Furthermore, Raman mapping revealed that in MGD meibum, the relative content of unsaturated lipids was markedly reduced, and the spatial distribution of saturated and unsaturated lipids was heterogeneous (Figures 3A–D).

### Changes in the relative content of symmetric and asymmetric lipids

**Figure 4:**
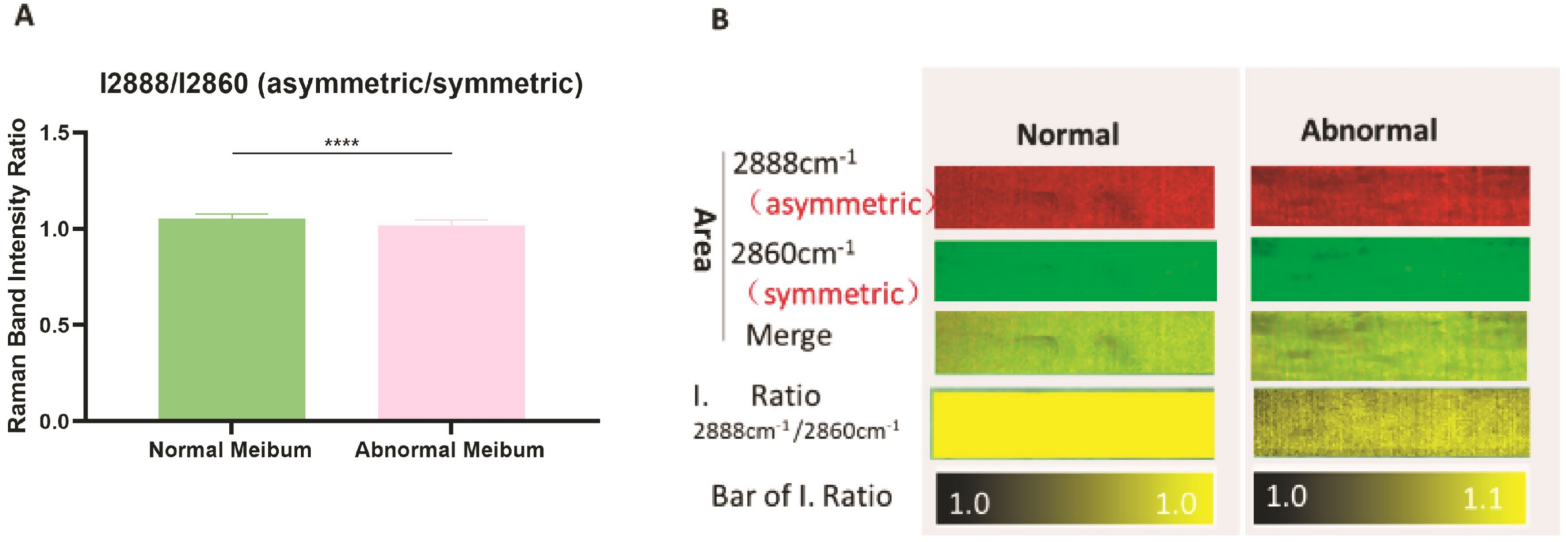
Relative contents of symmetric and asymmetric lipids in normal and MGD meibum. **(A)** Ratio of Raman band intensities I2888/I2860 cm^−1^ in normal and abnormal meibum. Data are presented as mean ± SD. Independent-samples *t*-test: Normal meibum (*n* = 20), 1.053538 ± 0.023517; abnormal meibum (*n* = 20), 1.01554 ± 0.030841; *p* < 0.0001. **(B)** Raman mapping analysis showing the spatial distribution of symmetric and asymmetric lipids.

Lipid molecules can be classified as symmetric or asymmetric according to their molecular structures. Liquid lipids are typically enriched in asymmetric molecules, whereas solid lipids contain more symmetric molecules. To investigate the relative content of these lipid types in MGD, we compared the Raman peak intensities at 2860 cm^−1^ (symmetric lipids) and 2,888 cm^−1^ (asymmetric lipids). The symmetric-to-asymmetric lipid ratio (I2888/I2860) differed significantly between MGD and normal meibum (*p* < 0.0001) (Figure 4A). Notably, although statistically significant, the mean values of the two groups were very close, suggesting that the actual magnitude of the ratio difference may be biologically small. Raman mapping revealed that symmetric and asymmetric lipids were uniformly distributed in healthy meibum, whereas in MGD meibum their spatial distribution became more heterogeneous (Figure 4B), similar to the patterns observed in Figures 3C,D, suggesting that subtle compositional changes may be accompanied by localized microstructural disruption.

### Changes in the relative content of proteins in MGD

**Figure 5:**
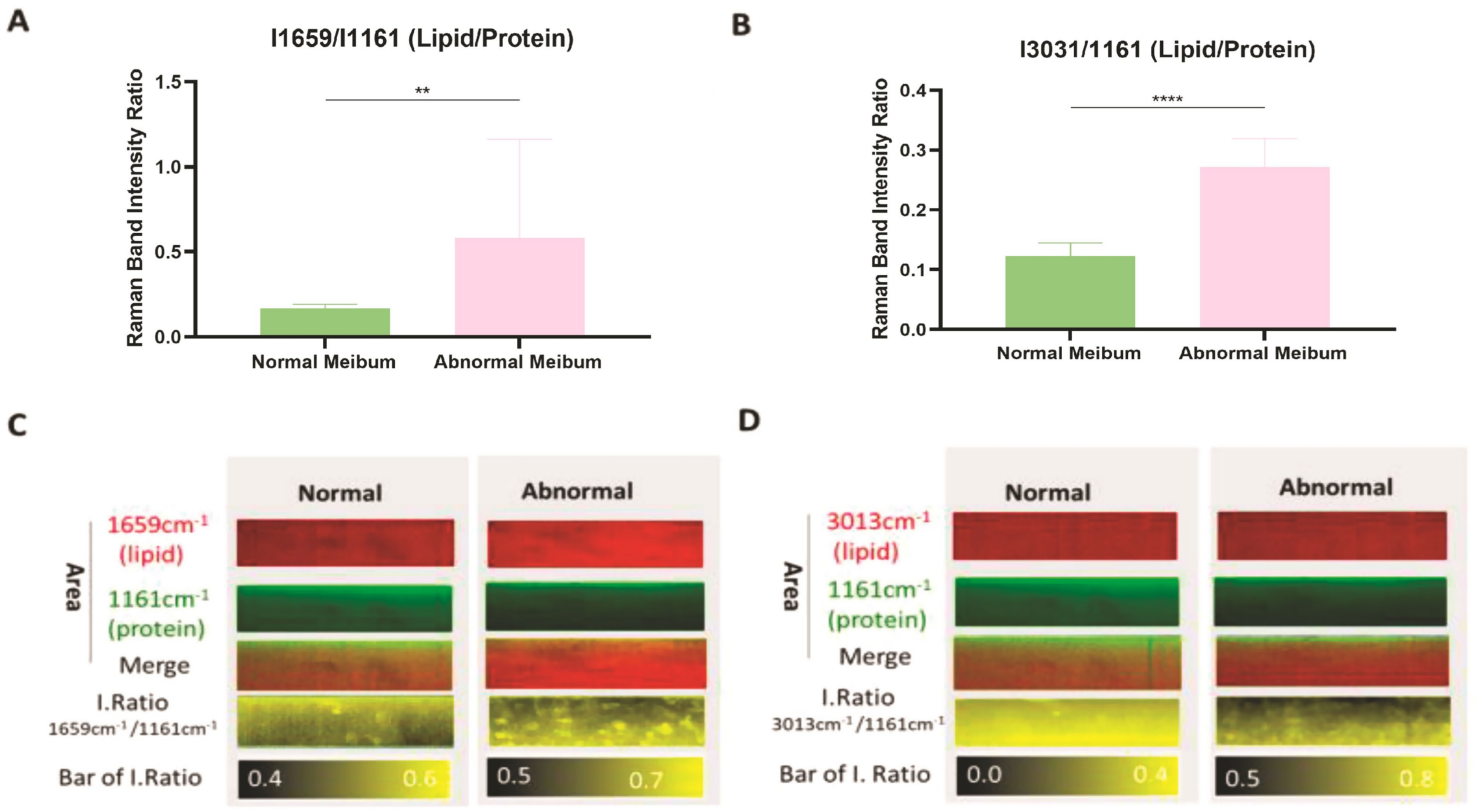
Relative contents of lipids and proteins in normal and MGD meibum. **(A)** Ratio of Raman band intensities I1659/I1161 cm^−1^ in normal and abnormal meibum. Data are presented as mean ± SD. Independent-samples *t*-test: Normal meibum (*n* = 20), 0.167166 ± 0.02414; abnormal meibum (*n* = 20), 0.580068 ± 0.58183; *p* = 0.0030 (*p* < 0.01). **(B)** Ratio of Raman band intensities I3013/I1161 cm^−1^ in normal and abnormal meibum. Data are presented as mean ± SD. Independent-samples *t*-test: Normal meibum (*n* = 20), 0.122912 ± 0.02228; abnormal meibum (*n* = 20), 0.271537 ± 0.048022; *p* < 0.0001. **(C,D)** Representative Raman mapping images showing the spatial distribution of lipid-to-protein ratios in normal and MGD meibum.

Proteins secreted by the meibomian gland are essential for maintaining the ocular surface microenvironment (25). Alterations in protein composition have been reported in MGD, and protein content also influences the flow properties of meibum. In our previous analyses, we observed a reduction in the relative content of unsaturated lipids in MGD. To further investigate protein-lipid balance, we compared the Raman peak intensities of unsaturated lipids (1,659 and 3,013 cm^−1^) with that of proteins (1,161 cm^−1^). The ratios I1659/I1161 and I3013/I1161 were significantly higher in MGD meibum than in normal meibum (*p* < 0.01) (Figures 5A,B), indicating a relative decrease in protein content in MGD. Consistent results were observed in Raman mapping analyses (Figures 5C,D), which also revealed heterogeneous distributions of proteins and lipids in MGD samples. These findings suggest that MGD is associated with reduced protein content and altered protein-lipid interactions in meibum.

## Discussion

We investigated the meibum of healthy individuals and MGD patients using Raman spectroscopy and imaging techniques. By analyzing lipid saturation, unsaturation, symmetry, asymmetry, and the ratios of Raman peak signals between lipids and proteins, we sought to elucidate potential pathological mechanisms of MGD. Based on a review of previous literature, Raman peaks in meibum were assigned to specific components: 1438 cm^−1^, 2,860 cm^−1^, and 2,888 cm^−1^ were attributed to saturated lipids, while 1,659 cm^−1^ and 3,013 cm^−1^ corresponded to unsaturated lipids. In meibum, fatty acids constitute the major lipid fraction, greatly exceeding hydrocarbons. Prior studies have primarily focused on the balance of saturated and unsaturated fatty acids in meibomian gland secretions; however, considerable controversy remains regarding their exact proportions.

From a molecular conformation perspective, a higher content of unsaturated fatty acids promotes better meibum flow properties (1, 26, 27). Unsaturated fatty acids contain a double bond between C9 and C10, and these C=C bonds are predominantly in the cis configuration, producing an ~30° bend in the hydrocarbon chain (28). This bending disrupts tight molecular packing, reduces van der Waals interactions, and lowers the melting point of fatty acids as the degree of unsaturation increases (28). Consequently, flow properties improve with increasing unsaturation (29). In contrast, saturated fatty acids are highly flexible and allow relatively free rotation around each C-C bond (30). Their conformational range is broad due to minimal steric hindrance between adjacent methylene groups, leading to a stable, extended conformation that is energetically favorable (31). The melting point of saturated fatty acids rises with increasing molecular weight.

Additionally, dietary lipids rich in unsaturated fatty acids, such as peanut oil and rapeseed oil, are typically liquid at room temperature, whereas those predominantly composed of saturated fatty acids, such as butter and lard, are solid (32). Thus, a higher proportion of unsaturated fatty acids contributes to increased fluidity of biological membranes.

Beyond their effects on meibum fluidity, unsaturated fatty acids also participate in inflammation and immune regulation (33). In contrast, saturated fatty acids are considered proinflammatory (34). Unsaturated fatty acids can be divided into monounsaturated fatty acids, which contain a single double bond, and polyunsaturated fatty acids, which contain two or more double bonds. Based on the position and function of the double bonds, polyunsaturated fatty acids are further classified into *ω*-6 species (such as linoleic and arachidonic acids) and ω-3 species (such as linolenic acid, DHA, and EPA). Unsaturated fatty acids form the structural skeleton of cell membranes, particularly in neuronal tissue, and play key roles in energy conversion and cellular signaling.

Recent studies have demonstrated systemic effects of ω-3 and ω-6 fatty acids, including maintaining membrane fluidity and normal cellular functions; esterifying cholesterol, thereby reducing circulating cholesterol and triglycerides; lowering blood viscosity and improving microcirculation; and enhancing neuronal activity (35). Polyunsaturated fatty acids also regulate systemic inflammation: ω-3 fatty acids generally exert anti-inflammatory effects, whereas ω-6 fatty acids may promote inflammation and amplify allergic responses (36).

The role of monounsaturated fatty acids in inflammation remains controversial. A study by Vaittinen et al. (37) reported that the relative abundance of saturated fatty acids was positively correlated with subcutaneous and visceral inflammation (except in the liver), while monounsaturated fatty acids were negatively correlated with inflammatory changes in adipose tissues. Similarly, Ralston et al. (38) demonstrated that saturated fatty acids activate the NLRP3 inflammasome, whereas both monounsaturated and polyunsaturated fatty acids inhibit its activity. Additional studies have shown that monounsaturated fatty acids may contribute to lowering blood glucose (39), regulating blood lipids (40), and reducing cholesterol levels (41).

Unsaturated fatty acids are also closely linked to immune regulation. Previous research has shown that the fatty acid composition of human immune cells can modulate their functional capacity. Hunsche et al. (42) demonstrated in a high-fat diet-induced obese mouse model that switching to a diet enriched in *ω*-3 polyunsaturated and monounsaturated fatty acids significantly improved chemotaxis, phagocytosis, digestive activity, natural killer cell activity, and lymphocyte proliferation. These results suggest that monounsaturated and *ω*-3 polyunsaturated fatty acids enhance immune responses and reduce oxidative stress (35).

Currently, research on the role of unsaturated fatty acids in the meibomian gland has primarily focused on the effects of dietary *ω*-3 supplementation in MGD (evaporative dry eye). However, the reported functions of ω-3 fatty acids remain controversial. Some studies suggest that ω-3 fatty acids reduce inflammatory cell activity by inhibiting NF-κB or competitively antagonizing ω-6 fatty acids, thereby alleviating inflammation (43). In addition, ω-3 supplementation has been reported to increase the number of triglyceride droplets and neutral lipid vesicles in meibomian gland epithelial cells, improve lipid quality, and alleviate glandular obstruction. Studies by Olenik et al. (44, 45), Malhotra et al. (46), and Bhargava et al. (47) also demonstrated that oral ω-3 intake improved tear film stability.

In contrast, a multicenter, double-blind clinical trial conducted by the International Dry Eye Assessment and Management Study Group found no significant differences between ω-3 supplementation and olive oil placebo in the treatment of moderate-to-severe dry eye disease. Neither group exhibited improvements in ocular surface disease index scores or ocular examination findings (48). Furthermore, no studies to date have specifically investigated the role of monounsaturated fatty acids in the pathophysiology of the meibomian gland.

Taken together, these findings indicate that unsaturated fatty acids not only influence meibum fluidity but also participate in the inflammatory and immune processes of the meibomian gland. Our study revealed that the relative content of unsaturated fatty acids in the meibum of patients with MGD is significantly reduced. Further investigations are required to clarify the precise contributions of unsaturated fatty acids to the inflammatory and immune regulation of meibomian gland function.

In addition to fatty acids, other unsaturated lipids such as squalene also appear to play important roles in meibomian gland physiology. Squalene, a highly unsaturated hydrocarbon compound, enhances superoxide dismutase activity and strengthens immune function. As a non-saponifiable lipid, it contributes to tear film stability, prevents tear saponification, and supports ocular surface health. It has been speculated that the foamy secretions observed during MGD, associated with lipid saponification in the eyelids, may result from a reduction in squalene levels.

In addition to changes in lipid saturation, our findings also revealed a statistically significant shift in the symmetric-to-asymmetric lipid ratio in MGD meibum. Although the absolute difference between groups was small, Raman mapping demonstrated increased spatial heterogeneity in MGD samples, suggesting that subtle conformational alterations in lipid organization may accompany compositional abnormalities. This structural imbalance may represent an additional dimension of meibum dysregulation beyond changes in lipid unsaturation.

Changes in the protein components of the meibomian gland are increasingly recognized for their impact on gland function. The lipid-protein mixture secreted by the meibomian gland maintains tear film permeability and stability while preventing evaporation. In this study, we found that the ratios of I1659/I1161 and I3013/I1161 in the meibum of patients with MGD were significantly higher than those in healthy individuals (*p* < 0.01), indicating a relative decrease in protein content in MGD meibum.

MGD is a major cause of dry eye and is clinically associated with tear film abnormalities and ocular surface inflammation, which can lead to irritation symptoms. In severe cases, it may cause corneal damage and impair visual function. Current evidence regarding protein alterations in MGD meibum includes a significant reduction in lipophilic proteins, which play important roles in MGD pathophysiology. Decreases in these proteins may impair the transport of free fatty acids (3) and hinder the clearance of toxic peroxidation metabolites, thereby triggering cell apoptosis (49, 50). Lipophilic proteins, normally abundant in the meibomian gland, also exhibit anti-inflammatory properties (51) and participate in cholesterol transport (52). These proteins have been reported to be significantly downregulated in MGD (53). In addition, ectodysplasin A (EDA) secreted by the meibomian gland promotes corneal epithelial cell proliferation and regulates epithelial barrier function (54–56). Studies have shown that mice with EDA gene mutations leading to meibomian gland developmental abnormalities, such as Tabby mice, exhibit corneal epithelial defects, stromal opacity, neovascularization, and ulceration (57).

Borchman and colleagues used infrared spectroscopy combined with principal component analysis to examine protein changes in meibum from healthy individuals and MGD patients. In contrast to our findings, they reported an increased protein-to-lipid ratio in MGD meibum. The authors speculated that proteins in meibum exhibit a linear relationship with lipid structure, molecular arrangement, and melting point. This discrepancy may be attributed to differences in sample collection, storage, or detection methods. Raman spectroscopy, compared with infrared spectroscopy, offers complementary advantages as it allows the detection of small sample volumes and provides accurate structural information even in the presence of water interference.

Although higher protein levels are expected to promote a more ordered lipid arrangement, increase viscosity, and raise the melting point, the reduced protein content observed in this study would theoretically favor improved meibum fluidity. However, meibum fluidity is influenced by multiple factors. Given that lipids account for over 90% of meibum, lipid-lipid interactions likely exert a greater influence on rheological properties than protein-lipid interactions. As a result, despite reduced protein levels, the meibum of patients with MGD remains viscous, granular, and toothpaste-like in consistency.

### Limitations and future directions

According to the 2023 Chinese expert consensus on meibomian gland dysfunction (20, 21), the disease can be classified into two main types based on meibum secretion patterns: hyposecretory MGD and hypersecretory MGD types. The hyposecretory MGD type is further subdivided into glandular atrophy and obstructive forms, with the obstructive type being the most common clinical presentation of MGD. In this study, conventional Raman spectroscopy was primarily applied to investigate compositional changes in meibum between healthy individuals and MGD patients with obstructive MGD.

However, several limitations of this study warrant further consideration:

#### Sample size and meibum classification

Based on physical characteristics, meibum can be categorized as clear and transparent, turbid, turbid-granular, or thick toothpaste-like in consistency. In the present study, only granular and toothpaste-like secretions were selected for analysis, and spectral signals were averaged. Future studies with larger sample sizes and separate analyses for each secretion type may yield more nuanced and valuable information. In addition, this study involved 40 samples (20 per group), which is comparable to some previous Raman spectroscopy studies of meibum. However, we acknowledge that the statistical power may be limited by this sample size. Larger, statistically powered cohorts will be essential to validate and generalize the present findings.

#### Hypersecretory (foamy) meibum

The hypersecretory type of meibum often appears foamy, which is associated with lipid saponification. Previous studies have suggested that a reduction in non-saponifiable lipids, such as squalene, is a major contributor to foamy secretions. Separate collection and analysis of this type of meibum could provide deeper insights into the pathophysiology of hypersecretory MGD.

#### Unsaturated fatty acids

In this study, the relative content of unsaturated lipids in meibum was significantly decreased. Prior research has shown that unsaturated fatty acids not only influence meibum fluidity and melting point but also play roles in inflammation and immune regulation. The therapeutic efficacy of orally administered polyunsaturated fatty acids, such as *ω*-3, in moderate-to-severe dry eye disease remains controversial. While some studies have suggested that monounsaturated fatty acids participate in inflammation and immune regulation in other tissues, their direct effects on meibomian gland tissues remain unclear. Elucidating the roles of both polyunsaturated and monounsaturated fatty acids in glandular inflammation and immune regulation could provide new insights into the mechanisms of MGD.

#### Protein composition

The types and functions of proteins secreted by the meibomian gland are being increasingly recognized. Although this study used Raman spectroscopy to assess relative protein content in meibum, it did not identify specific protein types. Future studies employing advanced proteomic approaches, such as relative and absolute quantitative mass spectrometry, label-free proteomics, or high-performance liquid chromatography-mass spectrometry, may provide more detailed insights into protein expression profiles and functional alterations in both healthy individuals and MGD patients.

## Data Availability

The raw data supporting the conclusions of this article will be made available by the authors, without undue reservation.
